# A case report of misdiagnosed fetal lung mass and review of the literature

**DOI:** 10.3389/fped.2022.1045037

**Published:** 2023-02-10

**Authors:** Zongyu Wang, Chang Xu, Taozhen He, Miao Yuan

**Affiliations:** West China Hospital, Sichuan University, Chengdu, China

**Keywords:** fetal lung interstitial tumor, pediatric lung tumor, fetal lung mass, diagnosis, surgery

## Abstract

The patient was a male neonate, and a prenatal ultrasound had detected a right lung mass. He was born at term and after delivery had tachypnea and feeding difficulties. A chest x-ray and a computed tomography (CT) scan revealed a large mass in the right chest with compression on the right lung after birth. We initially considered congenital pulmonary airway malformation (CPAM). After conservative treatment, his respiratory symptoms worsened gradually, and he required continuous supplemental oxygen. The symptoms could not be relieved by puncturing due to a postnatal ultrasound having shown a mass with anechoic microcystic spaces. He therefore underwent an emergency thoracotomy and lobectomy at 14 days of age. The pathology was consistent with fetal lung interstitial tumor (FLIT). The patient remained healthy at the three-month follow-up. We reviewed the literature on FLIT and found that, to date, 23 cases have been reported worldwide.

## Introduction

Fetal lung interstitial tumor (FLIT) is a newly-defined disease first reported by Dishop et al. (1, 2). Reading the literature on FLIT revealed that 23 cases have been reported worldwide. Of the 23 reported cases, 14 had an incorrect initial diagnosis, and 9 cases an ambiguous one. Shah et al. report that the initial diagnosis for one patient was congenital pulmonary airway malformation (CPAM) (3). Yoshida et al. report that one patient was initially considered to have CPAM or pulmonary sequestration (4). We can see that FLIT is easily misdiagnosed.

Misdiagnosis may increase clinical risk because the progression and treatment of FLIT differs from other conditions. Dishop et al. report that, in one case, FLIT was misdiagnosed as airway obstruction and did not receive surgery until doctors observed that the mass had increased (1). Prompt and accurate diagnosis of FLET can avoid both a delay in treatment and the administering of unsuitable treatment. However, the lack of a uniform standard at present leads to difficulties in diagnosing FLIT. We are therefore retrospectively analyzing a patient with FLIT who was transferred to our hospital, and reviewing the literature in order to summarize the characteristics of the disease with the aim that doctors might better diagnose FLIT.

## Case report

**Figure 1:**
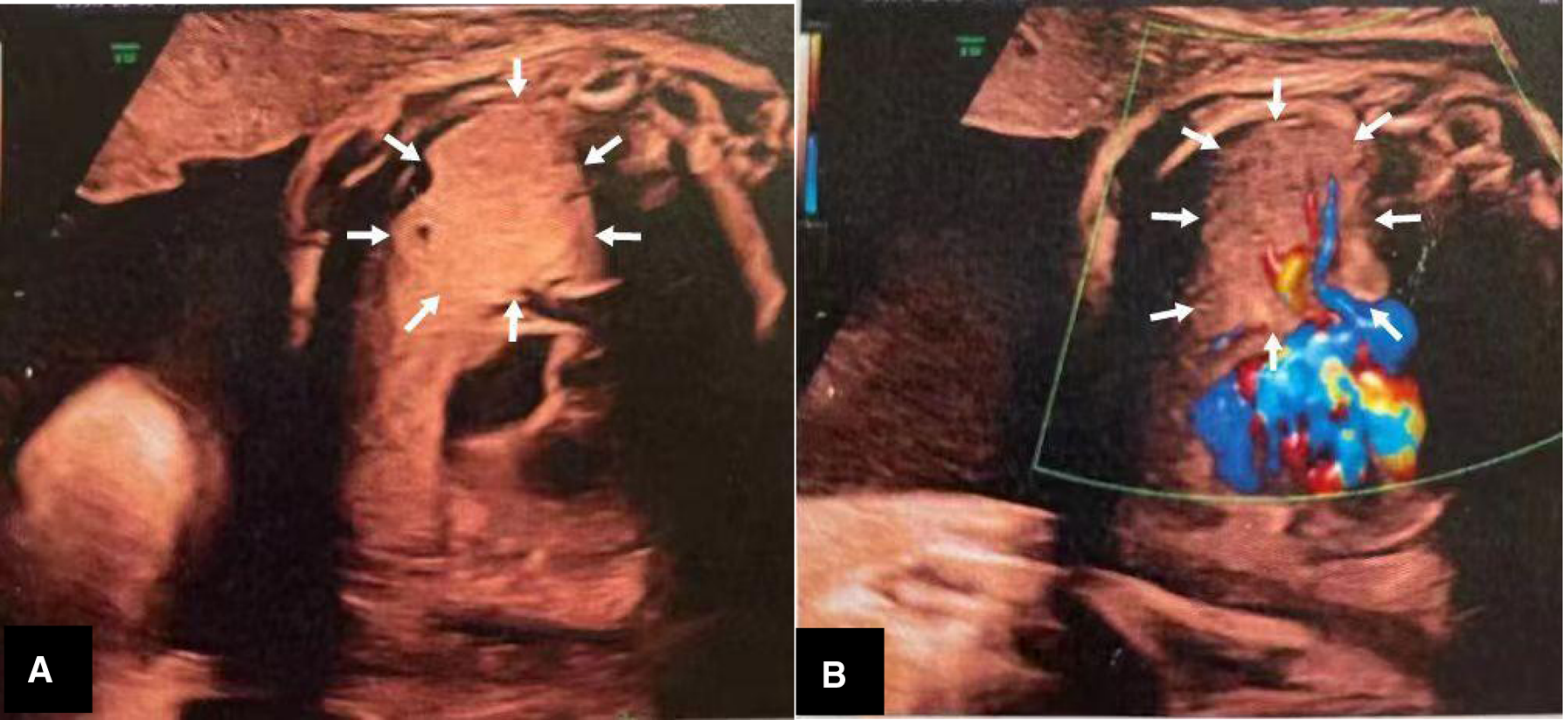
(**A**,**B**) Prenatal ultrasound showing a solid lung mass (arrows) with cystic components.

The patient was a male neonate (gestation, 37 weeks and 3 days; birth weight, 3,100 g). A prenatal ultrasound, at 33 weeks and 2 days of gestation, showed a solid, minimally hyperechoic, right-sided lung mass with cystic components, which suggested CPAM (Figure 1). The CPAM volume ratio (5) was 0.41 at 33 weeks, and 2.94 at 37 weeks of gestation. The pregnancy was otherwise uneventful. The patient was born at term by vaginal delivery at a local hospital. After delivery, he quickly developed tachypnea and was administered supplemental oxygen with a nasal catheter to stabilize his respiratory status. He also had feeding difficulties. At five hours of age he was transferred to our hospital for further evaluation and management.

**Figure 2:**
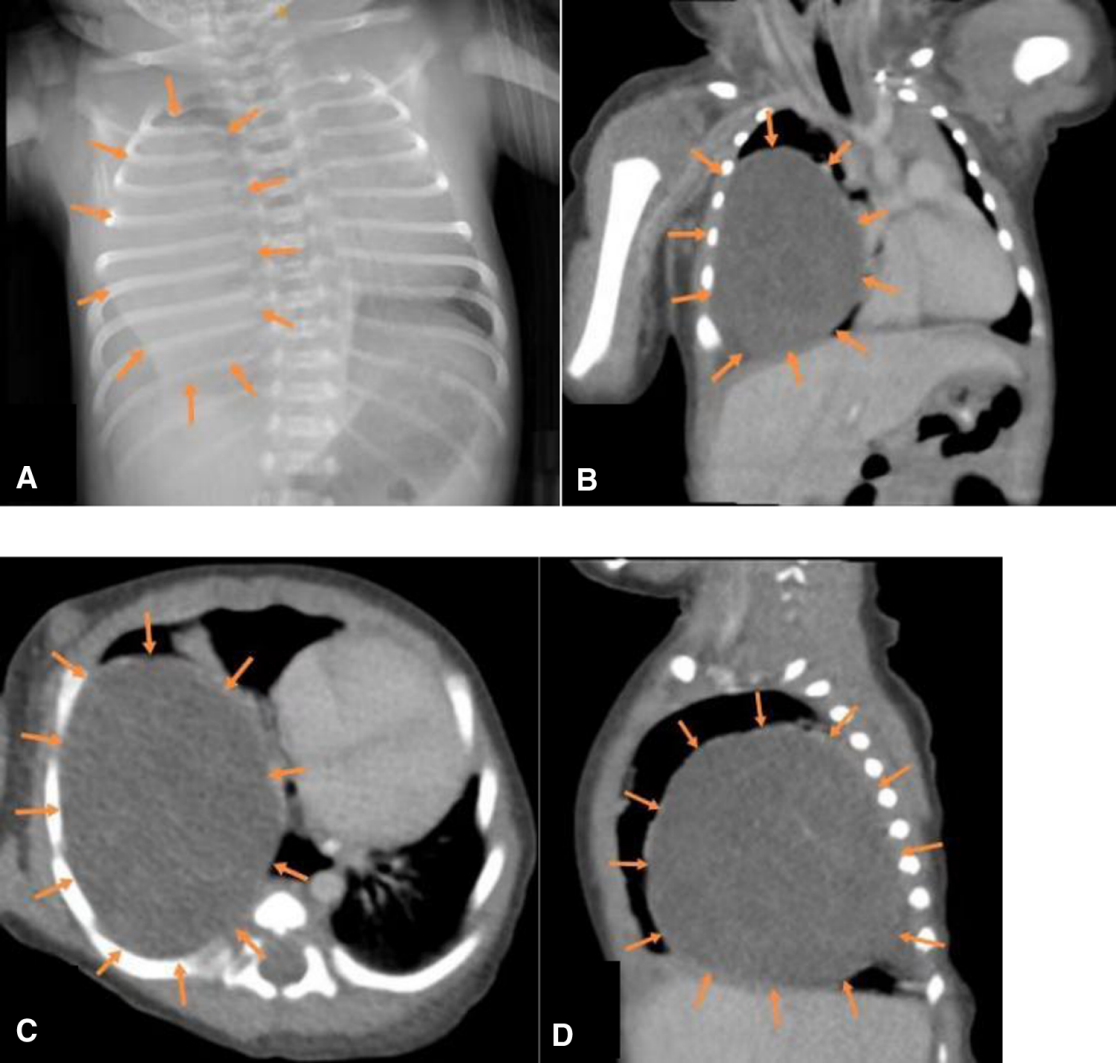
(**A**) x-ray and (**B–D**) CT (coronal, transverse and sagittal sections) showing a large mass in the right lower lobe.

**Figure 3:**
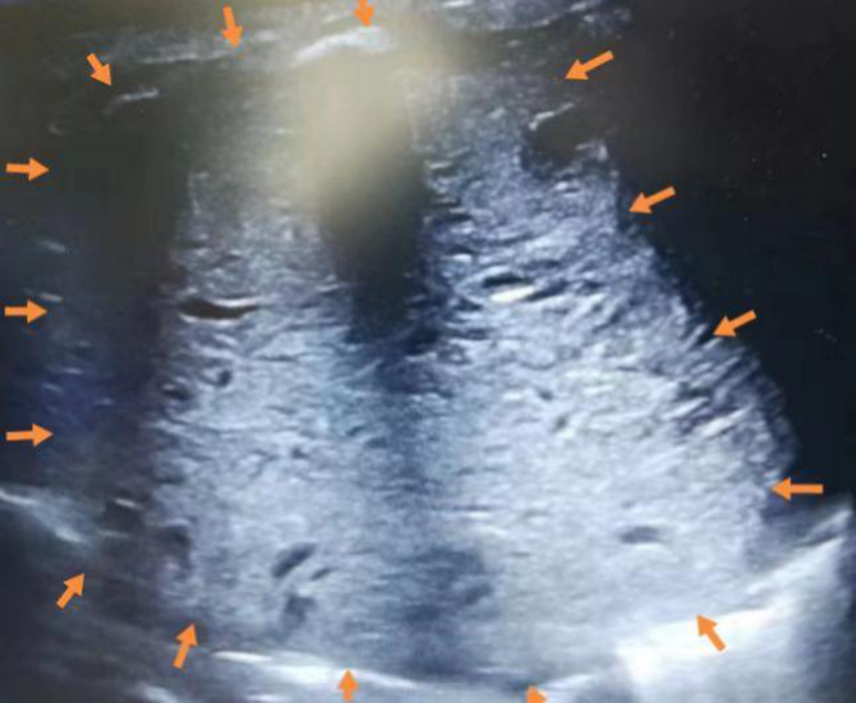
Postnatal ultrasound showing a well-circumscribed, isoechoic mass (arrows) (5.7 × 5.5 × 6.1 cm^3^) with anechoic cystic spaces.

On admission, the patient had tachypnea and decreased breath sounds. A chest x-ray and CT scan confirmed a large, solid mass with compression on the right lung (Figure 2). We initially considered CPAM. A postnatal ultrasound revealed a well-circumscribed, isoechoic mass (5.7 × 5.5 × 6.1 cm^3^) with anechoic cystic spaces (Figure 3). The patient underwent an emergency thoracotomy and lobectomy at 14 days of age.

**Figure 4:**
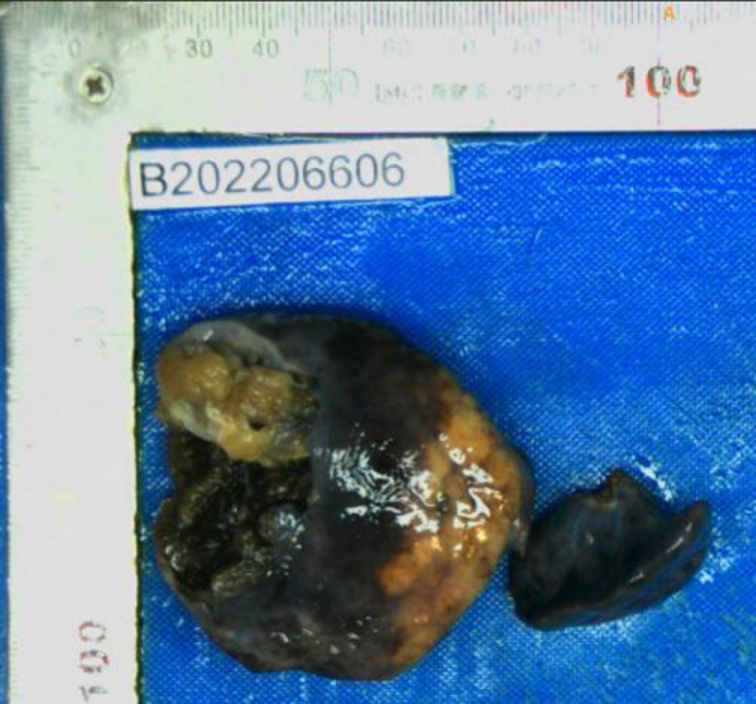
Macroscopic examination showing a spongy mass with cystic spaces.

During surgery, we found a large tumor in the right upper lobe with a mediastinal shift and so we performed a right upper lobectomy. The solid mass pressed on the airway, leading to abnormal an airway position. As a consequence, the right main bronchus was accidentally injured and reconstructed. The patient remained healthy approximately three months post-surgery with no adjuvant radiotherapy or chemotherapy. The excised specimen showed a fuscous, spongy, and well-circumscribed mass with cystic spaces (Figure 4). The pathology met the diagnostic criteria for FLIT.

## Discussion

Fetal lung interstitial tumor (FLIT) is easily misdiagnosed, especially as being CPAM (3, 4). In our case, the mass was misdiagnosed as CPAM and the doctors did not recognize the severe risk, resulting in the patient being born at a local hospital, rather than a specialist center. There are two main reasons for misdiagnosis. First, FLIT is rare, so doctors seldom consider this disease. Second, CPAM and FLIT have similar imaging manifestations. A prenatal ultrasound or magnetic resonance imaging (MRI) can detect FLIT. A prenatal ultrasound will show a solid, hyperechoic lung mass with or without cystic spaces (6, 7). After the prenatal ultrasound finds the lesion, an MRI can be performed for further evaluation. In our case the patient did not undergo an MRI because the price of an MRI is higher than that of an ultrasound, and there is no clear evidence to show that the additional information provided by an MRI influences the management of the patient. FLIT presents as a solid, well-circumscribed mass (1, 7), and the overall signal is slightly greater than that of the normal lung in a T2-weighted MRI (6). Some literature suggests that doctors should pay closer attention to the examination in the third trimester, in an effort to distinguish FLIT from CPAM. In CPAM, the maximum size appears at approximately 26–30 weeks of gestation (7–9), and the lesion may shrink or even disappear with the progression of pregnancy. However, in FLIT, the mass continues to grow and can lead to fetal edema in later pregnancy: Lazar et al. found a case of FLIT with fetal edema and heart failure at 37 weeks of gestation, which is later than CPAM would generally cause fetal edema (7).

**Figure 5:**
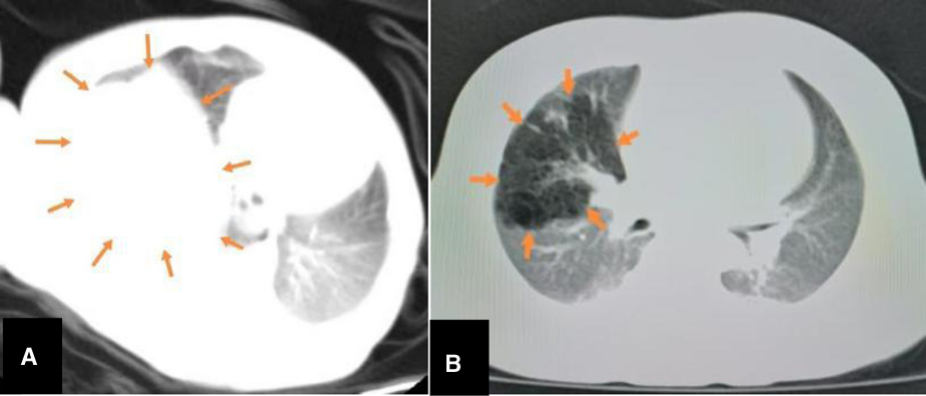
The comparison between CT features FLIT and CPAM. CT shows a mass (arrows) with uniform density in FLIT (**A**) and a mass (arrows) with low-density cystic spaces in CPAM (**B**).

Of the 23 reported cases, four were detected before birth, 18 were detected when symptoms developed after birth, and one was not described (Table 1). These figures demonstrate that doctors should remain vigilant for symptoms post-delivery. Most FLIT patients were born at term. All signs appeared within three months, and the most common symptom was respiratory difficulty (Table 1). Some patients also experienced feeding difficulties. Some patients with jaundice or a provisional diagnosis of dextrocardia are accidentally found to have a lung mass (3, 10). Our patient had also developed jaundice at nine days of age. Among these symptoms, feeding difficulties are relatively specific. A lung mass will generally lead to respiratory problems but not feeding problems (11). The reason that FLIT can lead to feeding difficulties may be that the lesion is large enough to compress the esophagus. The maximum diameter ranges from 2 cm to 9.5 cm, with the mean being 5.5 cm in FLIT (Table 1). A postnatal ultrasound and CT scan may also help in distinguishing between FLIT and CPAM. We find that the cystic content in FLIT is fluid, while the cystic content in CPAM is gas; so a postnatal ultrasound shows anechoic cystic spaces in FLIT (Figure 3), and a CT scan shows low-density cystic areas in CPAM (Figure 5).

**Table 1:** Summary of fetal lung interstitial tumor.

Author	Number of cases	Gender	Age at presentation	Initial symptoms	Tumor location	Tumor size (cm)	Treatment	Age at surgery	Gross features	Outcome
Dishop et al. 2010	10	Male (7); Female (3)	36 weeks of gestation–3 months postnatal	Respiratory distress; respiratory and feeding difficulties; respiratory syncytial virus bronchiolitis; diminished breath sounds; low-grade fever	RLL (4) LLL (2) RUL (2) LUL (1) RML (1)	2–7 (mean of 4.5)	Lobectomy (7); wedge excision (3); adjuvant chemotherapy(1)	37 weeks of gestation (EXIT)–5 months postnatal	Solid; soft, spongy, well-circumscribed mass	NER, follow-up varied from 9–182 months
de Chadarévian JP et al. 2011	1	Female	In utero	–	LLL	–	Lobectomy	Newborn	Gray-tan, well-circumscribed, soft, spongy mass with a fibrous capsule, and numerous cystic spaces	NER,84 months
Yoshida et al. 2013	1	Female	1d	Tachypnea	LLL	5	Lobectomy	13d	Solid; well-circumscribed mass with a thick capsule	NER,180 months
Onoda et al. 2014	1	Male	–	Mild apnea	LLL	2.6	Wedge resection	11d	Solid; well-circumscribed mass with equivalent cystic components	NER,36 months
Goto et al. 2015	1	Male	3d	Respiratory distress	–	3	Surgical resection	–	Solid; well-circumscribed and cystic mass	NER,24 months
Waelti et al. 2017	2	Male	0d	Respiratory distress	LUL	8.5	Lobectomy with incomplete resection	–	–	NER,36 months
		Male	33 weeks with fetal hydrops	Severe respiratory distress	RUL	9.5	Lobectomy	–	–	–
Phillips et al. 2019	1	–	26 weeks of twin gestation	Respiratory distress	LUL	6	Lobectomy	20d	Spongiform mass with cystic spaces	NER,1 months
Mocayar et al. 2019	1	Female	3d	Vomiting	RL	4.2	Surgical resection	–	–	–
Zhang et al. 2020	1	Female	7d	Yellowing of skin	RLL	6.5	Surgical resection	–	Gray-pink, soft spongy mass with an incomplete fibrous capsule	NER,14 months
Zhao et al. 2020	1	Male	0d	Tachypnea	RUL	6.5	Lobectomy	2d	Solid; spongy mass	NER,6 months
Shah et al. 2021	1	Male	0d	Provisional dextrocardia	LUL	6.5	Lobectomy	21d	Solid; soft, lobulated, tan-pink intraparenchymal mass with few cystic spaces	NER,12 months
Liu et al. 2021	1	Male	1d	Tachypnea	LLL	3.5	Lobectomy	–	Solid; dark-red, soft mass and a cyst with a diameter of 3.5 cm beside the mass	NER,9 months
Kuroda et al. 2021	1	Male	0d	Tachypnea and mild respiratory distress	RUL	8.5	Lobectomy	22d	Solid; spongy, well-circumscribed mass with a homogeneous surface and diffuse microcysts	NER,12 months
Present case, 2022	1	Male	33 weeks and 2 days of gestation	Tachypnea and feeding difficulties	RUL	6.2	Lobectomy	14d	Fuscous, spongy, well-circumscribed mass with cystic spaces	NER,3 months

RLL, right lower lobe; LLL, left lower lobe; RUL, right upper lobe; LUL, left upper lobe; RML, right middle lobe; RL, right lung; EXIT, Ex utero intrapartum treatment.

Tumor size (cm): maximum diameter.

NER, no evidence of recurrence.

A correct FLIT diagnosis can enable patients to receive timely and proper treatment. In the case of fetal edema, doctors can use hormones to try and relieve it. Phillips et al. report a case treated with betamethasone but that where polyhydramnios remained (12). Doctors might also perform an ex utero intrapartum treatment (EXIT) or early delivery. Lazar et al. report a fetus with fetal edema that received EXIT at 37 weeks gestation (7). Waelti et al. report that a patient with progressive fetal edema underwent an emergency cesarean section (13). When symptoms develop after birth, FLIT should be surgically resected as early as possible. The mean age at the time of surgery is 33 days in FLIT (Table 1). However, because the cystic content in CPAM is gas, in these cases percutaneous thoracic catheter drainage (PTCD) is an option (14). If symptoms ease, a patient with CPAM can receive watchful observation until their condition is improved and the likelihood of a positive outcome from thoracoscopic surgery increased.

Most FLIT cases received a lobectomy (Table 1). In our experience, doctors should pay close attention to the airway anatomy: we found that the solid mass put pressure on the airway, leading to an abnormal airway position. However, in CPAM there is less movement and deformity in anatomical structures. This may be because the cystic content and the size of the mass differ between FLIT and CPAM. Those patients with FLIT did not need radiotherapy or chemotherapy. The prognosis was good (15), and no recurrences were reported (Table 1). Waelti et al. report a case with a hilar remnant after surgery, this patient was followed up for three years and experienced no recurrence (13).

A pathological diagnosis cannot be confirmed by H&E staining alone, but with further specialist staining (16–18). To date, FLIT and pulmonary interstitial glycogenosis are the only diseases in which interstitial cells are rich in glycogen particles (4). In addition, the excised mass can be subjected to genetic testing to aid diagnosis (19). Zhao et al. report an initial pathological diagnosis of PPB in one case. However, no mutations were found in exons 24 and 25 of Dicer1, and so the final diagnosis was FLIT (12).

## Conclusion

FLIT is easily misdiagnosed. Prenatal ultrasound can aid diagnosis and should be performed regularly. An increasing mass volume in later gestation can indicate FLIT. After birth, feeding difficulties is a relatively specific symptom. In FLIT, a postnatal ultrasound shows a mass with anechoic microcystic spaces. CT scans usually show a mass with uniform density in FLIT, but a mass with low-density cystic areas in CPAM. Correct diagnosis can enable patients to receive timely and proper treatment. FLIT should be excised as early as possible, and most surgeons perform lobectomy. During surgery, surgeons should carefully monitor airway anatomy in order to avoid airway damage.

## Data Availability

The original contributions presented in the study are included in the article/Supplementary Material, further inquiries can be directed to the corresponding author.
